# Fluorescence Angiography with Dual Fluorescence for the Early Detection and Longitudinal Quantitation of Vascular Leakage in Retinopathy

**DOI:** 10.3390/biomedicines11020293

**Published:** 2023-01-20

**Authors:** Benjamin Pomeroy, Alexander W. Venanzi, Wei Li, Abigail S. Hackam, Midhat H. Abdulreda

**Affiliations:** 1 Diabetes Research Institute, University of Miami Miller School of Medicine, Miami, FL 33136, USA; 2 Department of Ophthalmology, University of Miami Miller School of Medicine, Miami, FL 33136, USA; 3 Cullen Eye Institute, Department of Ophthalmology, Baylor College of Medicine, Houston, TX 77030, USA; 4 Department of Surgery, University of Miami Miller School of Medicine, Miami, FL 33136, USA; 5 Department of Microbiology and Immunology, University of Miami Miller School of Medicine, Miami, FL 33136, USA

**Keywords:** diabetic retinopathy (DR), dual fluorescence, fluorescein angiography (FA), diabetic macular edema (DME), intravital imaging, longitudinal quantitation, noninvasive in vivo imaging, proliferative diabetic retinopathy (PDR), retinal vascular leakage

## Abstract

Background: Diabetic retinopathy (DR) afflicts more than 93 million people worldwide and is a leading cause of vision loss in working adults. While DR therapies are available, early DR development may go undetected without treatment due to the lack of sufficiently sensitive tools. Therefore, early detection is critically important to enable efficient treatment before progression to vision-threatening complications. A major clinical manifestation of early DR is retinal vascular leakage that may progress from diffuse to more localized focal leakage, leading to increased retinal thickness and diabetic macular edema (DME). In preclinical research, a hallmark of DR in mouse models is diffuse retinal leakage without increased thickness or DME, which limits the utility of optical coherence tomography and fluorescein angiography (FA) for early detection. The Evans blue assay detects diffuse leakage but requires euthanasia, which precludes longitudinal studies in the same animals. Methods: We developed a new modality of ratiometric fluorescence angiography with dual fluorescence (FA-DF) to reliably detect and longitudinally quantify diffuse retinal vascular leakage in mouse models of induced and spontaneous DR. Results: These studies demonstrated the feasibility and sensitivity of FA-DF in detecting and quantifying retinal vascular leakage in the same mice over time during DR progression in association with chronic hyperglycemia and age. Conclusions: These proof-of-concept studies demonstrated the promise of FA-DF as a minimally invasive method to quantify DR leakage in preclinical mouse models longitudinally.

## 1. Introduction

Diabetic retinopathy (DR) is a leading cause of vision loss in working adults. DR is caused by microvascular complications and neovascularization in the retina secondary to chronic exposure to diabetic hyperglycemia. Leakage of retinal vessels is an important early pathologic manifestation of DR that occurs before progression to more advanced complications associated with irreversible retinal damage that compromises vision. Early leakage in retinal vessels is typically widespread throughout the retina including in the periphery. If untreated, diffuse vascular leakage may progress to more localized leakage at focal lesions, leading to retinal thickness, diabetic macular edema (DME) or proliferative diabetic retinopathy (PDR), all of which threaten vision. Anti-angiogenic agents such as those against the vascular endothelial growth factor (VEGF) are used to treat various forms of DR, including DME and PDR [1,2]. As with any therapy, however, early intervention in the initial stages of DR would significantly increase the therapeutic efficacy of anti-angiogenic agents before significant retinal damage occurred. Therefore, it is critical to be able to detect early DR leakage in order to guide timely intervention to prevent advanced DR complications and ultimately preserve vision.

Animal models are widely used in DR research. Mice and rats remain the preferred models due to their quick reproduction rates, short lifespan, and small size, which allow studying disease progression within reasonable timeframes [3]. As early as 1963, Rakieten et al. demonstrated the induction of diabetes in rats and dogs using streptozotocin (STZ), which is a method commonly used today in research of diabetes and diabetes-related complications (e.g., DR) [4]. The pathology of DR in larger animals such as dogs more closely resembles that in humans. However, the disease progression is much slower and, therefore, rodent models remain the most utilized in preclinical studies despite the differences in some features of their DR pathology compared to humans [5]. Therefore, developing effective analytical tools for quantifying DR in mouse models is of major interest for testing new treatments for DR before transitioning them to clinical evaluation.

Noninvasive retinal imaging is a key diagnostic tool for evaluating DR. Optical coherence tomography (OCT) can detect changes in retinal thickness and DME but cannot detect vascular leakage [6]. OCT angiography (OCTA) can further detect occlusion and reperfusion in retinal vessels but has limited utility in quantifying vascular leakage [7]. Whereas fluorescein angiography (FA) can detect localized vascular leakage at focal lesions in the retina, conventional FA has poor sensitivity for reliably detecting and quantifying early diffuse leakage [8]. In contrast, terminal techniques using different fluorescence or radio-labeled tracers (e.g., Evans blue, FTIC-bovine serum albumin, or 125I-albumin) have the sensitivity to quantify diffuse leakage, but they are not applicable to humans, and they preclude longitudinal studies in the same research animals because they require whole body perfusion and euthanasia [9,10,11,12]. Therefore, there is a need for novel techniques that enable the noninvasive detection and longitudinal quantitation of diffuse retinal vascular leakage in both the clinical and research settings.

In this study, we developed a new modality of ratiometric fluorescence angiography with dual fluorescence (FA-DF) to reliably detect and longitudinally quantify diffuse leakage of retinal vessels. Conducting analyses in preclinical mouse models of induced and spontaneous DR, the current studies demonstrated the feasibility and sensitivity of FA-DF for detecting and quantifying diffuse leakage in retinas of the same mice over time during DR progression in association with chronic hyperglycemia and age. Therefore, this new noninvasive and longitudinal modality for detecting and quantifying retinal vascular leakage facilitates the preclinical investigation of DR and the development of therapeutics against it and has the potential to be further developed for clinical application.

## 2. Materials and Methods

### 2.1. Mice

C57BL/6 (B6; strain C57BL/6J; strain # 000664) and AKITA (strain C57BL/6-Ins2^Akita^/J; strain # 003548) mice were purchased from Jackson Laboratories (Bar Harbor, ME, USA) and housed under a 12/12 h of light/dark cycle with free access to food and water. B6 mice were made diabetic with streptozotocin (STZ; Sigma Aldrich; St. Louis, MO, USA) treatment as the induced DR model [13]. AKITA mice, which produce misfolded nonfunctional insulin, spontaneously became hyperglycemic within the first four weeks of life, developed progressive DR over time and were the spontaneous DR model [14,15]. Only heterozygous AKITA mice were used because homozygous mice die shortly after birth. In addition, only B6 and heterozygous AKITA males were used because of their higher susceptibility to DR [11]. All animal procedures in this study were reviewed and approved by the University of Miami’s Institutional Animal Care and Use Committee (IACUC; protocol # 20-133 approved on 24 August 2020).

### 2.2. Induced Model of DR

For diabetes induction, C57BL/6 (B6) mice were fasted overnight for 8 to 12 h and injected with a freshly prepared solution of STZ in 1x citrate buffer (Sigma Aldrich; St. Louis, MO, USA) at a single dose of 150 mg/kg of body weight [16]. Blood glucose levels were monitored daily after STZ injection to confirm hyperglycemia. Blood glucose was measured in a drop of blood collected from the tail vein using a portable digital glucometer (Contour Next; Bayer Healthcare LLC; Mishawaka, IN, USA). Mice with three consecutive glycemia readings ≥ 350 mg/dL were considered frankly diabetic. Diabetic mice were monitored thereafter three times per week for blood glucose levels and body weight until euthanasia. B6 mice made diabetic by STZ treatment were aged for 4 months to develop DR.

### 2.3. Evans Blue (EB) Assay

The EB assays were performed as previously described in detail [13]. In brief, the Evans blue dye (Sigma Aldrich; St. Louis, MO, USA) was injected intravenously into mice fully anesthetized by a 10:1 mixture ketamine and xylazine (100 and 10 mg/kg of body weight, respectively; ZooPharm; Laramie, WY, USA). The dye was circulated for 2.5 h to allow binding to albumin, and excess unbound dye was removed by perfusion with phosphate buffered saline (PBS; Sigma Aldrich; St. Louis, MO, USA). The mouse was then humanely euthanized, eyes were removed, and retinas isolated for ex vivo quantitation of Evans blue dye bound to leaked albumin in the extravascular space of the retina, as previously described [17].

### 2.4. Fluorescence Angiography with Dual Fluorescence (FA-DF)

FA-DF imaging was performed in mice under general anesthesia using a 10:1 ketamine–xylazine mixture as above with the eyes kept moist during imaging using balanced salt solution (BSS; Alkon Laboratories Inc; Fort Worth, TX, USA) to avoid corneal dryness. The pupils were dilated prior to anesthesia with tropicamide ophthalmic solution 0.5% (Akorn Inc.; Lake Forest, IL, USA). Baseline images were acquired in each mouse within 1 min before (nominal time zero) intravenous injection of a mixture of two fluorescence-labelled dextrans of different molecular weight (M.W.) at 5 mg/mL each in saline solution, and time-lapse fluorescence imaging of the central and peripheral retina vasculature was initiated immediately after repositioning the mouse on the stage of a clinical-grade Heidelberg Engineering Multiline HRA + OCT SN 2884 imaging system (Heidelberg Engineering Inc.; Franklin, MA, USA). We used 500-kDa fluorescein isothiocyanate (FITC)-labeled dextran (Cat # MFCD00132418; Sigma Aldrich; St. Louis, MO, USA) and 2000-kDa tetramethyl rhodamine (TRITC)-labeled dextran (Cat #D7139; ThermoFisher Scientific; Waltham, MA USA). In each mouse, images were acquired every 5 min after the injection for 30 min. Image pairs (1536 × 1636 pixels, 96 dpi, 24 bit) were acquired in the green and red channels by alternating between the appropriate instrument’s filter cubes (FA-PB for green and LWP542 for red) at every time-point. The same instrument settings (e.g., gain, exposure time, etc.) were used during each imaging session and throughout the longitudinal studies. After imaging, the mouse eyes were covered with a thin layer of antibiotic ophthalmic ointment (neomycin, polymyxin B sulfates, and dexamethasone; Bausch & Lomb Pharmaceuticals; Tampa, FL, USA) to prevent eye infection and drying of the cornea during recovery from anesthesia.

### 2.5. FA-DF Image Analysis

Time-lapse images acquired from each mouse were exported as tiff files and imported into Volocity software version 6.3.1 (Quorum Technologies Inc; Puslinch, ON, Canada), where the vascular leakage analysis was performed without image registration, as previously described [18]. In brief, blood vessels in the central and peripheral retina were automatically detected/selected in each channel by algorithms built into Volocity based on the green and red fluorescence, respectively. As before [18,19,20], the detection threshold was set to 3 to 5 times the standard deviation in the noise. Median fluorescence intensities (MFI) were measured independently in each channel inside and outside all blood vessels and capillaries captured in the entire image at every time-point after injection of the dyes. The outside to inside MFI ratio relative to time zero of the injection accounted for the leaked dye from blood vessels into the extravascular space (EVS) in every green/red image pair (i.e., for each dextran) acquired at 5, 10, 15, 20, 25, and 30 min after the injection. This was defined as the raw measure of “*Vascular Leakage*”.

## 3. Results

### 3.1. Detection of Dual Fluorescence Inside and Outside Blood Vessels of the Retina

**Figure 1:**
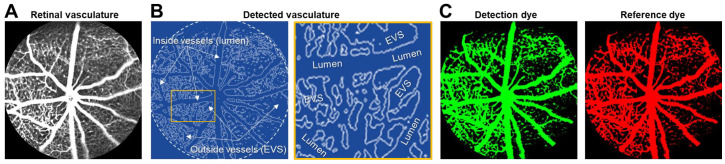
A novel method to noninvasively detect and quantify retinal vascular leakage with dual fluorescence in vivo. (**A**) Representative composite fundus image showing blood vessels in the central and peripheral retina where we performed the leakage analysis. (**B**) Autodetection of the vascular tree by Volocity software, as described in the Methods, which enables measuring of the mean fluorescence intensities (MFI) of the green and red fluorescence-labeled dextrans of the intravenously injected mixture inside the lumen of vessels and outside in the extravascular space (EVS). Shown on the right is a closeup of the area boxed in yellow highlighting the selection of the vessel lumen and EVS where the MFI measurements are obtained. (**C**) Representative fluorescence images of the retinal vascular tree in the green (FITC; left) and red (TRITC; right) channels, in which the MFI measurements inside and outside vessels of each dye are obtained to quantify the vascular leakage.

We performed initial studies to assess the feasibility of (a) acquiring composite fundus images (Figure 1A) with a conventional FA instrument (see Methods) and (b) identifying the intra- and extra-vascular spaces within the complete retinal vasculature (Figure 1B) after intravenous injection of a mixture of the fluorescent dyes (i.e., FITC and TRITC; 519 nm and 580 nm max emission, respectively) conjugated to dextrans with different M.W. Using our previous approach of vessel autodetection and selection in Volocity software [18,19,20], we successfully identified all vessels and the EVS among them in the central and peripheral retina (see closeup in Figure 1B). Further, we used intermediate and excessively large M.W. dextrans respectively conjugated to the green (FITC) and red (TRITC) dyes and were able to independently detect fluorescence emitted from each throughout the retinal vasculature without evidence of spectral overlap or interference between the channels (Figure 1C). As detailed below, the green-labeled intermediate M.W. dextran served as the leakage detection dye under pathological conditions, whereas the red-labeled large M.W. dextran served as a non-leaking reference, which allowed ratiometric measurements of the fluorescence for the leaked dye in the EVS relative to the non-leaked, remaining green dye in the vessel lumen at various time-points after injection (see below).

### 3.2. Longitudinal Quantitation of Retinal Vascular Leakage in the Induced DR Model of Diabetic C57BL/6 Mice

**Figure 2:**
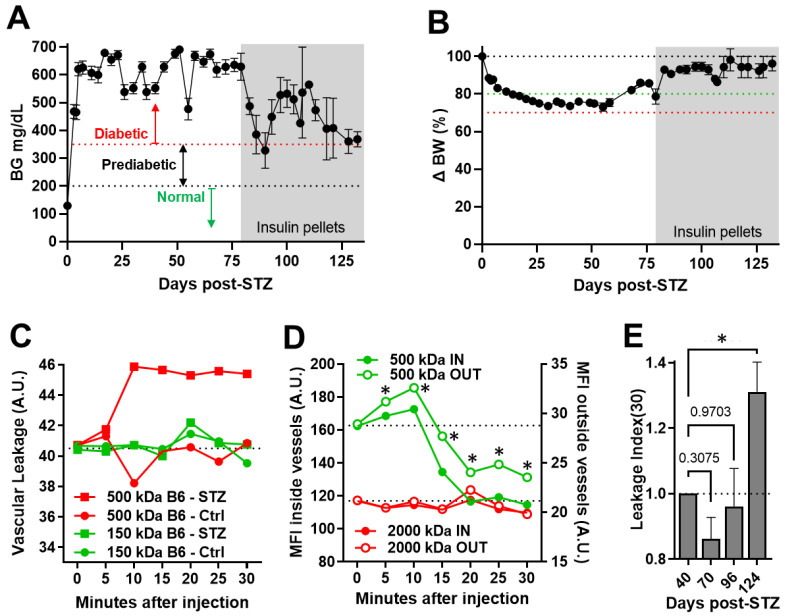
Longitudinal in vivo quantitation of retinal vascular leakage during the progression of induced DR in diabetic C57BL/6 mice. (**A**) Blood glucose levels in streptozotocin (STZ)-induced diabetic C57BL/6 mice showing chronic severe hyperglycemia, which leads to diabetic retinopathy (DR) in 4 months following diabetes induction. (**B**) Body weight changes (expressed as % delta; Δ) following diabetes induction before and during insulin therapy by slow-release insulin pellets (Linplant; LinShin Canada Inc; Toronto, Canada). Data are shown as means ± SEM (*n* = 3–15 mice). The animal numbers decreased overtime due to attrition in association with age and advanced diabetic complications. (**C**) Representative raw mean fluorescence intensity (MFI) measurements expressed in arbitrary units (A.U.) of 150-kDa (Sigma Cat # FD150S) and 500-kDa (Sigma Cat # 52194) dextrans conjugated to FITC and TRITC, respectively, in a STZ-diabetic (STZ) and a non-diabetic control (Ctrl) mouse. Measurements were obtained during 30 min after the injection, and shown are the MFI ratios outside to inside the vessels of the 150-kDa FITC-labeled (green lines) and 500-kDa TRITC-labeled (red lines) dextrans at each time point during the 30 min. Measurements were aligned to the same level at time 0 (immediately before injection) to highlight the dye’s accumulation over time in the EVS as a measure of the leaked dye (i.e., *vascular leakage*). (**D**) Representative raw MFI measurements of FITC-labelled 500-kDa (green) and TRITC-labeled 2000-kDa (red) dextrans inside (IN; left *Y*-axis) and outside (OUT; right *Y*-axis) retinal blood vessels of a diabetic AKITA mouse (* indicates significant difference between inside and outside with *p* value < 0.05 by two-tailed paired *t*-test). (**E**) *Leakage Index (30)* values obtained longitudinally in the same STZ-diabetic B6 mice at the indicated numbers of days after the STZ treatment. Data are shown as means ± SEM and plotted and analyzed in GraphPad Prism version 9.5.0 for Windows, GraphPad Software, San Diego, California USA, www.graphpad.com (*n* = 3–8 mice/time-point). Pairwise comparisons were performed by one-way ANOVA followed by Dunnett’s multiple comparison test of each time point compared to 40 days. Asterisk (*) denotes significance with *p* < 0.05, and numerical *p* values are shown when significance was not reached.

We performed longitudinal studies to assess the capability of FA-DF to detect and quantify the progression of induced DR in STZ-diabetic B6 mice. Diabetic mice were confirmed with severe hyperglycemia during the entire experiment (Figure 2A) and eventually needed assistance with insulin therapy to maintain their wellbeing (Figure 2A,B). Initially, studies were performed using 150-kDa FITC-labeled dextran as the leakage detection dye and 500-kDa TRITC-labeled dextran as the non-leaking reference. Similar parallel studies were also conducted in control non-diabetic B6 mice for comparison. These studies revealed that 150-kDa dextran leaked readily in both diabetic and non-diabetic mice and its fluorescence equilibrated inside and outside vessels within minutes after injection. By contrast, the 500-kDa dextran increased progressively in the EVS and reached a plateau at approximately 10 min after the injection in the diabetic mice (red symbols/lines) but not in the non-diabetic controls (Figure 2C), indicating that 500 kDa is a better choice for leakage detection than 150 kDa.

We next performed similar studies in diabetic AKITA mice using 500-kDa and 2000-kDa dextrans conjugated to FITC and TRITC as the leakage detection dye and non-leaking reference, respectively. These studies showed that the MFI of 500-kDa dextran initially increased in the EVS following the injection and progressively decreased after 10 min (Figure 2D; green lines open symbols); the MFI decreased inside the vessels as well (green lines filled symbols) due to clearance by the kidney as was routinely evidenced in the urine [19,21,22,23]. Notably, despite the progressive reduction of the 500-kDa dextran inside and outside the vessels after the initial peak around 10 min post-injection, significant differences in the MFI between the two compartments (inside and outside) were still measured throughout the 30 min after the injection. Moreover, these studies also showed that 2000-kDa dextran remained unchanged inside (red lines filled symbols) and outside (red lines open symbols) the vessels, further confirming its utility as a non-leaking reference even in these severely diabetic mice (Figure 2D).

Next, we defined the “*Leakage Index*”, which is a measure of the vascular leakage of 500-kDa dextran normalized to the non-leaking reference 2000 kDa dextran at any given time after the injection relative to time zero. Because it is a ratiometric measurement based on the leaked dye relative to the non-leaking reference, the leakage index is insensitive to signal drifts observed in the leakage detection channel in association with dye excretion in the urine or other in vivo imaging artifacts, such as breathing movement and transient lens clouding during imaging or cataract development over time. We used the leakage index at 30 min (“*Leakage Index (30)*”) to compare longitudinal changes in the retinal leakage of aged STZ-diabetic mice imaged using the mixture of 500-kDa and 2000-kDa dextrans at different times after the STZ treatment and during DR progression. The *leakage Index (30)* increased significantly in these diabetic B6 mice at 124 days after the STZ treatment (Figure 2E).

### 3.3. Longitudinal Quantitation of Retinal Vascular Leakage during the Progression of Spontaneous DR in AKITA Mice

**Figure 3:**
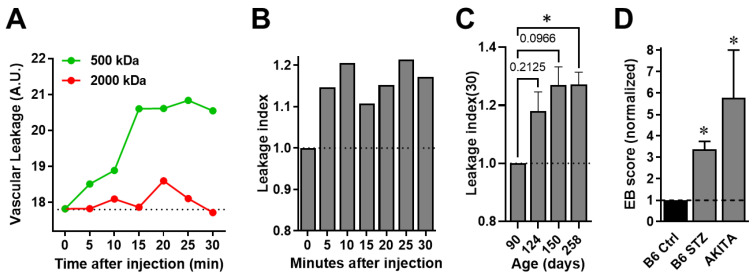
Detection and quantitation of DR progression by FA-DF in spontaneously diabetic AKITA mice in association with age. (**A**) Representative vascular leakage (arbitrary units; A.U.) of 500-kDa and 2000-kDa dextrans (conjugated to FITC and TRITC, respectively) and (**B**) *leakage index* values obtained at the indicated time-points during the 30 min post injection relative to time zero (before injection). The *vascular leakage* and *leakage index* values were obtained in an approximately 4month-old diabetic AKITA mouse based on ratiometric MFI measurements as described above. The FITC and TRITC-labeled dextrans were injected intravenously as a mixture in saline solution at 5 mg/mL each. (**C**) Longitudinal *Leakage Index (30)* values in the same AKITA mice at the indicated ages normalized to age 90 days, when the mice had been already diabetic for approximately 2 months. Data are shown as means ± SEM and were plotted and analyzed in GraphPad Prism (*n* = 3–5 mice/time-point). Asterisk (*) denotes significance with *p* < 0.05, and numerical *p* values are shown when significance was not reached. Pairwise comparisons were performed by one-way ANOVA followed by Dunnett’s multiple comparison test at each time point (age) compared to age 90 days. (**D**) Evans blue (EB) assay permeability scores showing retinal vascular leakage in association with spontaneous DR in ≥ 250 days old AKITA mice and in STZ-diabetic B6 (B6 STZ) mice ≥ 120 days after STZ treatment in comparison to non-diabetic B6 controls (B6 Ctrl). EB assays performed in dedicated cohorts different than those in the longitudinal studies. Data were normalized to the B6 Ctrl and are shown as means ± SEM (*n* = 12 mice/condition). Asterisk (*) denotes significant difference to the controls with *p* < 0.05 by *t*-test.

To further demonstrate the capabilities of FA-DF in detecting and quantifying diffuse vascular leakage in vivo and longitudinally, we performed additional studies in AKITA mice to assess the retinal vascular leakage as DR progressed spontaneously in these diabetic mice in association with age. Similar to the studies in the STZ-induced model, we used 500-kDa FITC-dextran for leakage detection and 2000-kDa TRITC-dextran as the non-leaking reference. Vascular leakage measurements of both dyes confirmed retinal leakage of the 500-kDa but not the 2000-kDa dextran (Figure 3A). Ratiometric leakage index measurements showed consistent retinal leakage during the 30 min after injection in these diabetic AKITA mice at age 124 days when they have been diabetic for approximately 60 days (Figure 3B); albeit significant differences in comparison to time zero were not measured at this age. However, extended follow-up in the same mice showed progressively increased *leakage index (30)* in association with DR progression as the animals aged, and a significant difference was measured at 258 days of age in comparison to when they were 90 days old (Figure 3C).

To confirm these and the above FA-DF findings on the retinal vascular leakage in both mouse models, we used EB assays to quantify the vascular leakage in ≥ 250 days old AKITA mice and STZ-diabetic B6 mice ≥ 120 days after STZ treatment. Consistent with the FA-DF findings, these EB analyses confirmed significant retinal vascular leakage in both models in comparison to non-diabetic B6 controls (Figure 3D).

### 3.4. Quantifying the Change in the Caliber of Retinal Vessels in Longitudinal FA-DF Images during DR Progression

**Figure 4:**
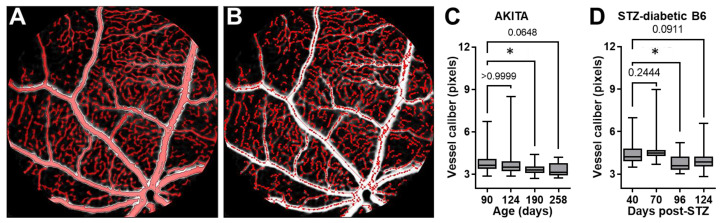
Changes in retinal vessel caliber during DR progression detected and quantified in longitudinal FA-DF studies. (**A**) Representative fundus image showing automatic detection and selection of retinal vessels in Volocity software based on the green fluorescence intensity in the analysis of vascular caliber (diameter). (**B**) Outline of the “skeleton” of the retinal vascular tree corresponding to the retinal vasculature detected/selected in A that was used to measure the “skeletal diameter” (vessel caliber) of all identified “objects” (vessels) within the image. (C and D) Longitudinal measurements of vessel caliber in (**C**) the diabetic AKITA mice from age 90 to 258 days (*n* = 3–5 mice/time-point) and (**D**) STZ-diabetic B6 mice at 40, 70, 96, and 124 days after STZ treatment (*n* = 3–8 mice/time-point). Caliber measurement units are shown in pixels since the tiff images were not calibrated for pixel size. Data are shown as Box and Whiskers with error bars representing the minima and maxima. Horizontal lines inside the boxes represent the median. Data were plotted and analyzed in GraphPad Prism. Pairwise comparisons were performed by one-way ANOVA followed by Dunnett’s multiple comparison test at each time point compared to the earliest time point in each series. Asterisk (*) denotes significance with *p* < 0.05, and numerical *p* values are shown when significance was not reached. Loss of significance at the latest time points in both models was likely due to reduced numbers of mice in association with age and increased attrition due to advanced diabetic complications.

It has been established that reduced caliber (diameter) and narrowing of retinal vessels occurs in association with DR [24,25]. To further corroborate the above FA-DF findings on retinal vascular leakage and further demonstrate additional benefits of longitudinal imaging studies by this noninvasive method, we assessed changes in the caliber of retinal vessels in longitudinal images acquired in the same diabetic AKITA and B6 mice of the above studies. We performed the vascular caliber (diameter) analysis according to our previous description [19,26]. In brief, automatic detection/selection of retinal vessels was done using built-in object selection algorithms in Volocity software. The selection was based on the green fluorescence intensity in images using a threshold of 1 to 3 times the standard deviation of the peak of noise signal in the first images acquired after injecting the mixed dyes in each mouse during the repeated imaging sessions in the longitudinal studies. Local contrast adjustment to correct for spectral aberration at the edge of vessels (vascular wall) and a noise removal step with a “fine filter” were included before measuring the vessel diameter (Figure 4A). Following the objects’ selection, the “skeleton” of the vascular tree was then automatically generated by the software (Figure 4B), based on which the “skeletal diameter” (vessel caliber) measurements were returned for all identified “objects” (vessels) within the image, including the central and peripheral retina. The analysis was performed on longitudinal images acquired in the same mice without image registration since we did not intend to measure changes in individual vessels. The results, however, showed a significantly reduced median caliber of retinal vessels in association with DR progression and the above-measured increases in the retinal vascular leakage in both mouse models (Figure 4C,D).

## 4. Discussion

In this study, we developed a new technique of ratiometric FA-DF to quantify vascular leakage in the diabetic retina. Conventional FA uses only one dye, fluorescein-sodium, with a low M.W. (0.376 kDa) allowing it to diffuse freely through microcapillaries even in non-pathological conditions. This results in high background fluorescence signal, low signal-to-noise ratio, and poor sensitivity to reliably detect diffuse retinal leakage in early DR development. Conventional FA is also qualitative and does not allow quantitation of leakage, although, attempts have been made to quantify retinal permeability in FA images [27]. Currently, the EB assay is the gold-standard method for quantifying retinal vascular leakage in preclinical DR models, especially rodents, but it requires animal perfusion and euthanasia and, therefore, precludes longitudinal studies in the same animals. Other research groups have evaluated the permeability (leakage) of retinal vessels and dye clearance noninvasively by dual-tracer FA using fluorescein-sodium (0.376 kDa) and another low M.W. fluorescence-labeled tracer (70-kDa dextran) [28]. However, both tracers leaked freely, which severely limits the spatial and temporal resolution to reliably detect and quantify diffuse DR leakage. In addition, the studies by Russ and colleagues were not conducted longitudinally. Thus, the combined ready leakage of the dual, low molecular-weight tracers and non-longitudinal nature of these studies hindered the assessment of disease progression in the same animals over time.

In contrast, the findings presented here demonstrated that FA-DF allows the longitudinal assessment of retinal vascular leakage in the same mice. The use of ratiometric measurements of the two fluorescent dyes also achieved reliable in vivo quantitation of early diffuse retinal leakage in two mouse models of induced and spontaneous DR. FA-DF measurements revealed a significant increase in retinal vascular leakage at time points consistent with the onset of DR in both models [13,17]. By using a semi-permeable FITC-dextran (500-kDa) for leakage detection and a non-leaking reference (2000-kDa TRITC-dextran), we acquired ratiometric measurements and longitudinally quantified changes in dye leaked out of retinal vessels in the same diabetic mice as their DR progressed. This dual dye ratiometric approach allowed for reliable quantitation of the fluorescence intensity inside and outside blood vessels even in conditions with a high background signal. Notably, fluorescence ratiometric analysis is routinely used to quantify free intracellular calcium handling in single cells but, to our knowledge, ratiometric measurements have never been used previously to quantify vascular leakage in the setting of retinopathy. Ratiometric measurements are relatively insensitive to instrumental and biological drifts and other in vivo imaging influences like breathing movement, transient corneal clouding, cataract and chromatic aberration. They are also insensitive to differences in refractive power of eye structures at the excitation wavelengths utilized here. This is because if any signal drifts are to occur, they will be uniform for both dyes, and this will not impact the measured ratio since it is a function of the leaking dye only. As such, ratiometric measurements are superior in reliability and sensitivity to quantify small changes in fluorescence without the need for image registration or alignment in longitudinal time-lapse studies, as demonstrated here. Importantly, noninvasive FA-DF studies in the same live animals circumvent the combined limitations of the invasive EB assay and the poor resolution of conventional FA and enable the longitudinal assessment of retinal vascular leakage in association with diabetes and other retinopathies.

Despite its common use in the clinical setting, the qualitative and quantitative limitations of conventional FA are well recognized and, as such, the use of OCT/OCTA has become more common in ophthalmic practice. OCT and OCTA can reliably detect changes in retinal thickness, DME, PDR, as well occlusion/reperfusion in vessels of the retina; however, they cannot detect vascular leakage [6,7]. Other methods by confocal or multi-photon microscopy to achieve high-resolution in vivo imaging of blood vessels and their complications deep within tissues, such as in the retina, have also been pursued [18,19,21,22,23,29,30,31,32,33,34]. Research groups have investigated by multi-photon microscopy the permeability of blood vessels in the skin [35,36]. We previously assessed vascular leakage under inflammatory conditions using in vivo confocal microscopy in pancreatic islets and kidney glomeruli as well [18,19]. Indeed, our experience in these studies provided the foundation of the current FA-DF analyses in the diabetic retina. However, multi-photon microscopy may not be applicable to the clinical setting for the assessment of DR leakage due to the requirement for high energy Class IIIB lasers that pose serious risk of heat-damage to live tissues [37], which will unfortunately limit the potential utility of such high spatial-resolution modalities in patients. By contrast, the FA-DF technique reported here uses low-energy excitation wavelengths (e.g., 488 nm) from less-powered light sources (e.g., light emitting diodes or Class I lasers) without the risk of photo-damage to the retina or other tissues. The fluorescence-labelled dextrans are also safe for use in humans and the utilized FA imaging system (Heidelberg Spectralis SN 2884) is routinely applied in ophthalmic clinical practice. Therefore, with further development, FA-DF has the potential to be adapted to the clinical setting.

As demonstrated in the above presented findings, FA-DF enabled the detection and quantitation of retinal vascular leakage at various stages of DR in the employed mouse models. Future studies could also demonstrate its utility in other animal models. Vascular leakage occurs in DR and other retinal pathologies and is the result of increased permeability of blood vessels and compromised integrity of the blood–retina barrier, a structure that functions as a selective filter between blood vessels of the retina and the rest of the body vasculature. In general, the permeability of blood vessels depends on different factors, including the shape and size (i.e., M.W.) of molecules and the vascular wall fenestration, which changes with inflammation and vascular endothelial cell pathologies [35,36]. In the setting of DR, the blood–retina barrier is also compromised by chronic hyperglycemia via several pathways, including the accumulation of advanced glycation end products (AGEs), activation of protein kinase C, and the conversion of glucose to sorbitol and fructose through the polyol metabolic pathway, among other mechanisms [38]. One of the most detrimental consequences of compromised retinal vasculature is the formation of diabetic macular edema (DME), which could occur at any DR stage and is the most common cause of vision loss in diabetic patients [39]. Prior to the development of DME, decreased integrity of the blood–retina barrier and increased permeability of the retinal vasculature both lead to the accumulation of subretinal fluid. This in turn leads to capillary ischemia, hyperglycemia-induced oxidative stress, and associated increase in VEGF production that leads to neovascularization and chronic inflammation, among other factors that together can compromise the physical integrity of the retina and its function [40]. Notably, the increase in vascular permeability (i.e., leakage) is one of the earliest pathologic features of DR pathogenesis. Thus, detection of vascular leakage provides one of the earliest points to identify the initiation of DR pathogenesis and can provide significant opportunities for early medical intervention to prevent further retinal damage and advanced complications that can lead to vision loss, which currently occurs often in patients with DR. Our studies demonstrated that longitudinal FA-DF is feasible in the same diabetic mice and can detect and quantify early retinal vascular leakage and its progression. Thus, FA-DF could facilitate significantly to the preclinical development and assessment of therapeutics for the early prevention or treatment of DR.

Moreover, the current report demonstrated the relatively ready availability of FA-DF for preclinical studies and highlighted its utility as a minimally invasive intravital imaging method with superior capabilities compared to conventional FA. This also underscored its potential utility in the clinical setting. Although, future studies will be required to evaluate its sensitivity in detecting and assessing changes in retinal vascular leakage in DR patients with and without treatments. If proven successful in DR, FA-DF will also be applicable to other vasculopathies characterized by leakage, such as wet age-related macular degeneration (AMD) and central retinal vein occlusion [41]. Furthermore, with the recent efforts incorporating computational tools and artificial intelligence in the field of ophthalmology to assist in the detection of DR, glaucoma, AMD, and other ophthalmic conditions [42,43,44], the future incorporation of such powerful tools to FA-DF will streamline the image analysis in a fashion more compatible with its application at point-of-care locations. Though, FA-DF will also have certain limitations in clinical application consistent with those of conventional FA in patients who are pregnant or with kidney failure [45]. There are also potential concerns about allergic reactions to the dyes conjugated to injected dextrans [46]. But with proper diligence in examining patient drug allergies and any disclosed prior allergic reactions to FA, FA-DF could prove safe and effective in detecting and quantifying early diffuse DR leakage to assess disease progression or treatment efficacy. Other practical considerations that could hinder the clinical applicability of FA-DF, such the length of the procedure, should also be considered. While the optimal timeframe for FA-DF in the clinical setting is currently unknown, the above preclinical studies suggest that it could be similar to that of conventional FA, lasting for 10 to 30 min. Notably, given that FA-DF could enable the detection of early retinal leakage that current intravital imaging tools cannot (e.g., FA, OCT, and OCTA), FA-DF could complement the clinical arsenal to improve the early diagnosis of DR and other retinopathies.

Finally, we established the *Leakage Index* as a new ratiometric parameter to compare changes in the leakage of retinal vessels over time. We used here the leakage index at 30 min after injection of the labeled dextrans “*Leakage Index (30)*” to assess the progression of DR longitudinally in the same mice (Figure 2E and Figure 3C). We chose *Leakage Index (30)* since it was the latest time point after the injection and when the leakage detection dye (500-kDa FITC-dextran) unambiguously reached a steady state in the EVS in the diabetic AKITA mice (Figure 3A). However, the leakage index values also increased relative to the baseline throughout the 30 min after injection, albeit not significantly until the mice reached >258 days of age (Figure 3B,C). Similarly, there were significant differences in the MFI signal of the same dye inside and outside the vessels, starting at 5 min after the injection in the diabetic B6 mice (Figure 2D). Consequently, these results suggest that FA-DF imaging sessions with shorter timeframes could possibly reveal significant changes in retinal leakage in larger cohorts. Therefore, future studies are warranted to examine these and other aspects of FA-DF such as assay sensitivity, signal window, and reproducibility across other DR models in other species and to optimize the analysis parameters for its wider adoption.

## 5. Conclusions

In conclusion, our novel minimally invasive approach of FA-DF enables the detection of diffuse retinal vascular leakage and its quantitative assessment during DR progression in two preclinical mouse models of induced and spontaneous DR. These findings demonstrate that DA-DF could address a significant unmet need for preclinical DR research relying on rodent models, which are less prone to advanced forms of DR complications (e.g., PDR and DME) that are more amenable for investigation by existing noninvasive modalities like FA, OCT, and OCTA. Our studies also demonstrate that FA-DF enables the longitudinal quantitation of DR leakage in the same animals. Thus, FA-DF may prove a valuable tool in the preclinical development and assessment of DR therapeutics. Future studies will also evaluate its feasibility and potential utility in the clinical setting to aid in the early diagnosis of DR and the assessment of treatment efficacy in conjunction with other imaging modalities available in the clinical toolkit.

## Data Availability

All data related to this study are reported herein.

## References

[1] Arrigo A., Aragona E., Bandello F. (2022). VEGF-targeting drugs for the treatment of retinal neovascularization in diabetic retinopathy. Ann. Med..

[2] Vaziri K., Schwartz S.G., Relhan N., Kishor K.S., Flynn H.W. (2015). New Therapeutic Approaches in Diabetic Retinopathy. Rev. Diabet. Stud..

[3] Olivares A.M., Althoff K., Chen G.F., Wu S., Morrisson M.A., DeAngelis M.M., Haider N. (2017). Animal Models of Diabetic Retinopathy. Curr. Diab. Rep..

[4] Rakieten N., Rakieten M.L., Nadkarni M.V. (1963). Studies on the diabetogenic action of streptozotocin (NSC-37917). Cancer Chemother. Rep..

[5] Engerman R.L., Bloodworth J.M. (1965). Experimental Diabetic Retinopathy in Dogs. Arch. Ophthalmol..

[6] Lang G.E. (2007). Optical coherence tomography findings in diabetic retinopathy. Dev. Ophthalmol..

[7] Chua J., Sim R., Tan B., Wong D., Yao X., Liu X., Ting D.S.W., Schmidl D., Ang M., Garhöfer G. (2020). Optical Coherence Tomography Angiography in Diabetes and Diabetic Retinopathy. J. Clin. Med..

[8] Hui F., Nguyen C.T., Bedggood P.A., He Z., Fish R.L., Gurrell R., Vingrys A.J., Bui B.V. (2014). Quantitative spatial and temporal analysis of fluorescein angiography dynamics in the eye. PLoS ONE.

[9] Fukumura D., Xu L., Chen Y., Gohongi T., Seed B., Jain R.K. (2001). Hypoxia and acidosis independently up-regulate vascular endothelial growth factor transcription in brain tumors in vivo. Cancer Res..

[10] LeBlanc M.E., Wang W., Ji Y., Tian H., Liu D., Zhang X., Li W. (2019). Secretogranin III as a novel target for the therapy of choroidal neovascularization. Exp. Eye Res..

[11] Barber A.J., Antonetti D.A., Kern T.S., Reiter C.E., Soans R.S., Krady J.K., Levison S.W., Gardner T.W., Bronson S.K. (2005). The Ins2Akita mouse as a model of early retinal complications in diabetes. Invest. Ophthalmol. Vis. Sci..

[12] Williamson J.R., Chang K., Tilton R.G., Prater C., Jeffrey J.R., Weigel C., Sherman W.R., Eades D.M., Kilo C. (1987). Increased vascular permeability in spontaneously diabetic BB/W rats and in rats with mild versus severe streptozocin-induced diabetes. Prevention by aldose reductase inhibitors and castration. Diabetes.

[13] Li W., Webster K.A., LeBlanc M.E., Tian H. (2018). Secretogranin III: A diabetic retinopathy-selective angiogenic factor. Cell Mol. Life Sci..

[14] Han Z., Guo J., Conley S.M., Naash M.I. (2013). Retinal angiogenesis in the Ins2(Akita) mouse model of diabetic retinopathy. Invest. Ophthalmol. Vis. Sci..

[15] Yoshioka M., Kayo T., Ikeda T., Koizumi A. (1997). A novel locus, Mody4, distal to D7Mit189 on chromosome 7 determines early-onset NIDDM in nonobese C57BL/6 (Akita) mutant mice. Diabetes.

[16] Wu K.K., Huan Y. (2008). Streptozotocin-induced diabetic models in mice and rats. Curr. Protoc. Pharmacol..

[17] Rong X., Tian H., Yang L., Li W. (2019). Function-first ligandomics for ocular vascular research and drug target discovery. Exp. Eye Res..

[18] Abdulreda M.H., Molano R.D., Faleo G., Lopez-Cabezas M., Shishido A., Ulissi U., Fotino C., Hernandez L.F., Tschiggfrie A., Aldrich V.R. (2019). In vivo imaging of type 1 diabetes immunopathology using eye-transplanted islets in NOD mice. Diabetologia.

[19] Kistler A.D., Caicedo A., Abdulreda M.H., Faul C., Kerjaschki D., Berggren P.O., Reiser J., Fornoni A. (2014). In vivo imaging of kidney glomeruli transplanted into the anterior chamber of the mouse eye. Sci. Rep..

[20] Miska J., Abdulreda M.H., Devarajan P., Lui J.B., Suzuki J., Pileggi A., Berggren P.O., Chen Z. (2014). Real-time immune cell interactions in target tissue during autoimmune-induced damage and graft tolerance. J. Exp. Med..

[21] Almaça J., Molina J., Arrojo E.D.R., Abdulreda M.H., Jeon W.B., Berggren P.O., Caicedo A., Nam H.G. (2014). Young capillary vessels rejuvenate aged pancreatic islets. Proc. Natl. Acad. Sci. USA.

[22] Abdulreda M.H., Faleo G., Molano R.D., Lopez-Cabezas M., Molina J., Tan Y., Echeverria O.A., Zahr-Akrawi E., Rodriguez-Diaz R., Edlund P.K. (2011). High-resolution, noninvasive longitudinal live imaging of immune responses. Proc. Natl. Acad. Sci. USA.

[23] Nyqvist D., Speier S., Rodriguez-Diaz R., Molano R.D., Lipovsek S., Rupnik M., Dicker A., Ilegems E., Zahr-Akrawi E., Molina J. (2011). Donor islet endothelial cells in pancreatic islet revascularization. Diabetes.

[24] Lombardo M., Parravano M., Serrao S., Ducoli P., Stirpe M., Lombardo G. (2013). Analysis of retinal capillaries in patients with type 1 diabetes and nonproliferative diabetic retinopathy using adaptive optics imaging. Retina.

[25] Bek T. (2017). Diameter Changes of Retinal Vessels in Diabetic Retinopathy. Curr. Diab. Rep..

[26] Boden J., Wei J., McNamara G., Layman H., Abdulreda M., Andreopoulos F., Webster K.A. (2012). Whole-mount imaging of the mouse hindlimb vasculature using the lipophilic carbocyanine dye DiI. Biotechniques.

[27] Allen C.L., Malhi N.K., Whatmore J.L., Bates D.O., Arkill K.P. (2020). Non-invasive measurement of retinal permeability in a diabetic rat model. Microcirculation..

[28] Russ P.K., Gaylord G.M., Haselton F.R. (2001). Retinal vascular permeability determined by dual-tracer fluorescence angiography. Ann. Biomed. Eng..

[29] Sharma R., Yin L., Geng Y., Merigan W.H., Palczewska G., Palczewski K., Williams D.R., Hunter J.J. (2013). In vivo two-photon imaging of the mouse retina. Biomed. Opt. Express.

[30] Stremplewski P., Komar K., Palczewski K., Wojtkowski M., Palczewska G. (2015). Periscope for noninvasive two-photon imaging of murine retina in vivo. Biomed. Opt. Express.

[31] Palczewska G., Golczak M., Williams D.R., Hunter J.J., Palczewski K. (2014). Endogenous fluorophores enable two-photon imaging of the primate eye. Invest. Ophthalmol. Vis. Sci..

[32] Palczewska G., Dong Z., Golczak M., Hunter J.J., Williams D.R., Alexander N.S., Palczewski K. (2014). Noninvasive two-photon microscopy imaging of mouse retina and retinal pigment epithelium through the pupil of the eye. Nat. Med..

[33] Das T., Payer B., Cayouette M., Harris W.A. (2003). In vivo time-lapse imaging of cell divisions during neurogenesis in the developing zebrafish retina. Neuron.

[34] Bremer D., Pache F., Günther R., Hornow J., Andresen V., Leben R., Mothes R., Zimmermann H., Brandt A.U., Paul F. (2016). Longitudinal Intravital Imaging of the Retina Reveals Long-term Dynamics of Immune Infiltration and Its Effects on the Glial Network in Experimental Autoimmune Uveoretinitis, without Evident Signs of Neuronal Dysfunction in the Ganglion Cell Layer. Front. Immunol..

[35] Egawa G., Nakamizo S., Natsuaki Y., Doi H., Miyachi Y., Kabashima K. (2013). Intravital analysis of vascular permeability in mice using two-photon microscopy. Sci. Rep..

[36] Ono S., Egawa G., Kabashima K. (2017). Regulation of blood vascular permeability in the skin. Inflamm. Regen..

[37] Alexander N.S., Palczewska G., Stremplewski P., Wojtkowski M., Kern T.S., Palczewski K. (2016). Image registration and averaging of low laser power two-photon fluorescence images of mouse retina. Biomed. Opt. Express.

[38] Whitehead M., Wickremasinghe S., Osborne A., Van Wijngaarden P., Martin K.R. (2018). Diabetic retinopathy: A complex pathophysiology requiring novel therapeutic strategies. Expert Opin. Biol. Ther..

[39] Romero-Aroca P., Baget-Bernaldiz M., Pareja-Rios A., Lopez-Galvez M., Navarro-Gil R., Verges R. (2016). Diabetic Macular Edema Pathophysiology: Vasogenic versus Inflammatory. J. Diabetes Res..

[40] Wang W., Lo A.C.Y. (2018). Diabetic Retinopathy: Pathophysiology and Treatments. Int. J. Mol. Sci..

[41] Scheppke L., Aguilar E., Gariano R.F., Jacobson R., Hood J., Doukas J., Cao J., Noronha G., Yee S., Weis S. (2008). Retinal vascular permeability suppression by topical application of a novel VEGFR2/Src kinase inhibitor in mice and rabbits. J. Clin. Invest..

[42] Abràmoff M.D., Lavin P.T., Birch M., Shah N., Folk J.C. (2018). Pivotal trial of an autonomous AI-based diagnostic system for detection of diabetic retinopathy in primary care offices. NPJ Digit. Med.

[43] Wen J.C., Lee C.S., Keane P.A., Xiao S., Rokem A.S., Chen P.P., Wu Y., Lee A.Y. (2019). Forecasting future Humphrey Visual Fields using deep learning. PLoS ONE.

[44] Peng Y., Dharssi S., Chen Q., Keenan T.D., Agrón E., Wong W.T., Chew E.Y., Lu Z. (2019). DeepSeeNet: A Deep Learning Model for Automated Classification of Patient-based Age-related Macular Degeneration Severity from Color Fundus Photographs. Ophthalmology.

[45] Chen R., Liang A., Yao J., Wang Z., Chen Y., Zhuang X., Zeng Y., Zhang L., Cao D. (2022). Fluorescein Leakage and Optical Coherence Tomography Angiography Features of Microaneurysms in Diabetic Retinopathy. J. Diabetes Res..

[46] Meira J., Marques M.L., Falcão-Reis F., Rebelo Gomes E., Carneiro Â. (2020). Immediate Reactions to Fluorescein and Indocyanine Green in Retinal Angiography: Review of Literature and Proposal for Patient’s Evaluation. Clin. Ophthalmol..

